# Fully automatic algorithm for detecting and tracking anatomical shoulder landmarks on fluoroscopy images with artificial intelligence

**DOI:** 10.1007/s00330-023-10082-8

**Published:** 2023-08-11

**Authors:** Eleonora Croci, Hanspeter Hess, Fabian Warmuth, Marina Künzler, Sean Börlin, Daniel Baumgartner, Andreas Marc Müller, Kate Gerber, Annegret Mündermann

**Affiliations:** 1 Department of Biomedical Engineering, University of Basel, Basel, Switzerland; 2 Department of Orthopaedics and Traumatology, University Hospital Basel, Basel, Switzerland; 3 School for Biomedical and Precision Engineering, University of Bern, Bern, Switzerland; 4 IMES Institute of Mechanical Systems, Zurich University of Applied Sciences, Winterthur, Switzerland; 5 Department of Clinical Research, University of Basel, Basel, Switzerland

**Keywords:** Shoulder, Rotator cuff injuries, Fluoroscopy, Artificial intelligence, Motion

## Abstract

**Objective:**

Patients with rotator cuff tears present often with glenohumeral joint instability. Assessing anatomic angles and shoulder kinematics from fluoroscopy requires labelling of specific landmarks in each image. This study aimed to develop an artificial intelligence model for automatic landmark detection from fluoroscopic images for motion tracking of the scapula and humeral head.

**Materials and methods:**

Fluoroscopic images were acquired for both shoulders of 25 participants (*N* = 12 patients with unilateral rotator cuff tear, 6 men, mean (standard deviation) age: 63.7 ± 9.7 years; 13 asymptomatic subjects, 7 men, 58.2 ± 8.9 years) during a 30° arm abduction and adduction movement in the scapular plane with and without handheld weights of 2 and 4 kg. A 3D full-resolution convolutional neural network (nnU-Net) was trained to automatically locate five landmarks (glenohumeral joint centre, humeral shaft, inferior and superior edges of the glenoid and most lateral point of the acromion) and a calibration sphere.

**Results:**

The nnU-Net was trained with ground-truth data from 6021 fluoroscopic images of 40 shoulders and tested with 1925 fluoroscopic images of 10 shoulders. The automatic landmark detection algorithm achieved an accuracy above inter-rater variability and slightly below intra-rater variability. All landmarks and the calibration sphere were located within 1.5 mm, except the humeral landmark within 9.6 mm, but differences in abduction angles were within 1°.

**Conclusion:**

The proposed algorithm detects the desired landmarks on fluoroscopic images with sufficient accuracy and can therefore be applied to automatically assess shoulder motion, scapular rotation or glenohumeral translation in the scapular plane.

**Clinical relevance statement:**

This nnU-net algorithm facilitates efficient and objective identification and tracking of anatomical landmarks on fluoroscopic images necessary for measuring clinically relevant anatomical configuration (e.g. critical shoulder angle) and enables investigation of dynamic glenohumeral joint stability in pathological shoulders.

**Key Points:**

• *Anatomical configuration and glenohumeral joint stability are often a concern after rotator cuff tears.*

• *Artificial intelligence applied to fluoroscopic images helps to identify and track anatomical landmarks during dynamic movements.*

• *The developed automatic landmark detection algorithm optimised the labelling procedures and is suitable for clinical application.*

**Supplementary Information:**

The online version contains supplementary material available at 10.1007/s00330-023-10082-8.

## Introduction

Rotator cuff tears are often associated with glenohumeral joint instability [1–3]; however, no consistent pattern of scapular motion has been described [4]. During abduction, patients have been found to have more upward scapular rotation [5–11], normal scapular rotation [12, 13] or even a decrease in upward rotation [14]. Similarly, no conclusive findings have been made about glenohumeral translation [3, 15–18]. A consistent method for assessing shoulder kinematics would therefore provide clear understanding and consequently be beneficial for patient treatment.

Various methods have been suggested for assessing glenohumeral biomechanics. In the past, mainly conventional radiographs — imaging the shoulder in different arm positions — were used to assess the glenohumeral joint [1, 2, 15, 16, 19, 20]. However, kinematics is likely to differ between static and dynamic conditions, and hence recently, 3D-to-2D model to image registration techniques have been introduced for assessing shoulder kinematics during scapular plane abduction [13, 21–26]. While this method has high accuracy, it requires computed tomography (CT) images, hence exposing patients to high ionising radiation [27]. More recently, another method for estimating glenohumeral translation with single-plane fluoroscopy, requiring the labelling of five landmarks [28], has been suggested, but measurements of scapular rotation or critical shoulder angle [29] should also be possible. This method is associated with low radiation exposure and has good reliability with manual labelling [28]. However, because this process is time consuming, its applicability is limited on a large scale.

At present, artificial intelligence is gaining application in medical imaging. Deep neural networks have been used on magnetic resonance images (MRI) for classifying rotator cuff tears [30] and segmenting rotator cuff muscles [31, 32] and the glenohumeral joint [32, 33]. Deep learning has been reported also for quantifying and characterising rotator cuff muscle degeneration from CT scans [34]. While on radiographs, deep learning has been employed to detect humerus fractures [35], and to classify shoulder implants [36]. For measurement of the critical shoulder angle, artificial intelligence has also been used for landmark detection from radiographic images with accurate, reproducible and rapid measurements [37].

This study aimed to develop an artificial intelligence model for automatic landmark detection on fluoroscopic images during a 30° arm abduction and adduction movement in the scapular plane. This model aims to provide quick assessment of anatomical configuration and kinematics of the shoulder and is expected to efficiently yield accurate and reproducible results based on prior artificial intelligence applications.

## Materials and methods

### Participants

**Fig. 1:**
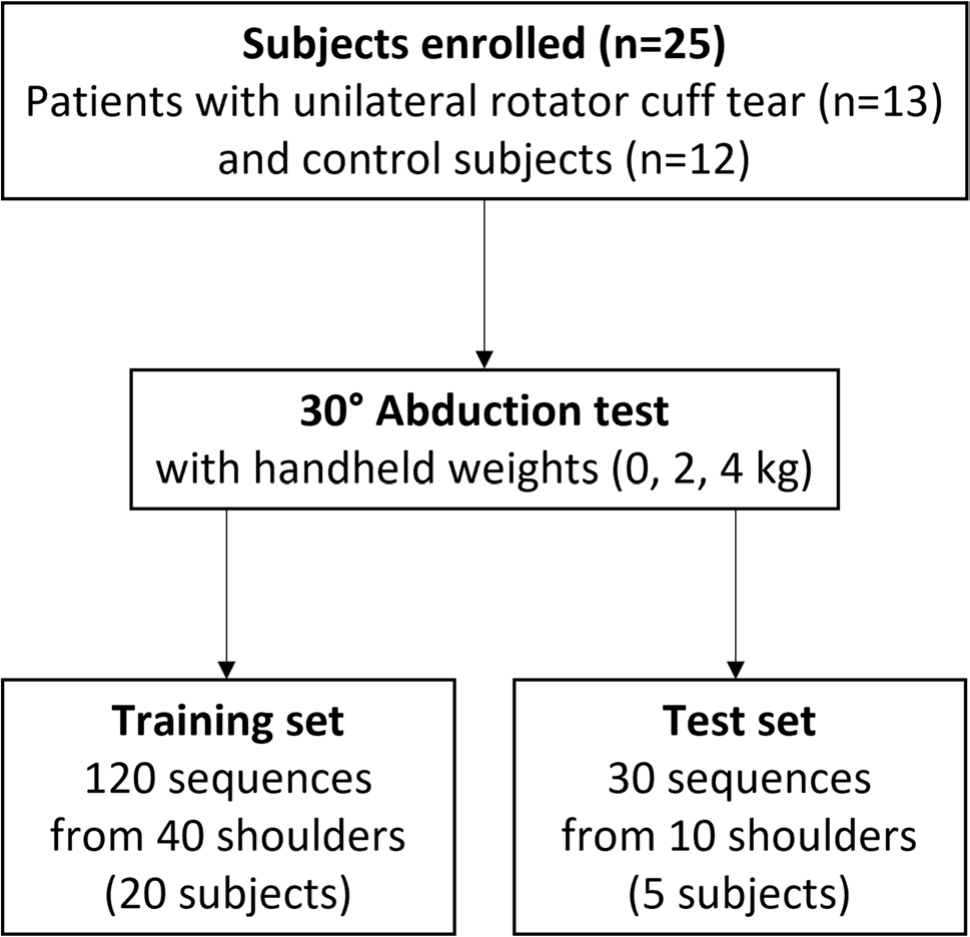
Flowchart of the study

This prospective study is part of an umbrella study on glenohumeral biomechanics [38] that was approved by the regional ethics board and conducted in accordance with the Declaration of Helsinki. Participants were enrolled between May 2021 and January 2023. Exclusion criteria were as follows: BMI > 35 kg/m^2^; inability to provide informed consent; prior operative treatments of the upper extremity; clinical history of the glenohumeral joints (only of the contralateral side for patients); and neuromuscular disorders affecting upper limb movements or other pathologies influencing shoulder joint mobility. Informed consent was obtained from all participants included in the study. Figure 1 depicts the study design with the cohort for the training and test sets.

### Image acquisition

Single-plane fluoroscopy images (Multitom Rax, Siemens Healthineers) were acquired for all shoulders during a 30° loaded and unloaded shoulder abduction test in the scapular plane. Testing procedures were performed as described in the study of Croci et al [28]. Participants abducted both arms simultaneously in the scapular plane up to 30°, first without handheld weight and then with 2-kg and 4-kg additional handheld weight in a randomised order. To maintain a comparable movement rate, verbal commands were given to the participants. Images were captured first for the right shoulder and then for the left shoulder with a pulse rate of 3 Hz (in 13 subjects) and 10 Hz (in 12 subjects). Image resolution was 946 × 958 pixels with a pixel size of 0.35 × 0.35 mm. The exact pixel dimensions were calibrated with a reference sphere (Ø = 25 mm) placed in the field of view of the fluoroscopy images.

### Automatic landmark detection algorithm

**Fig. 2:**
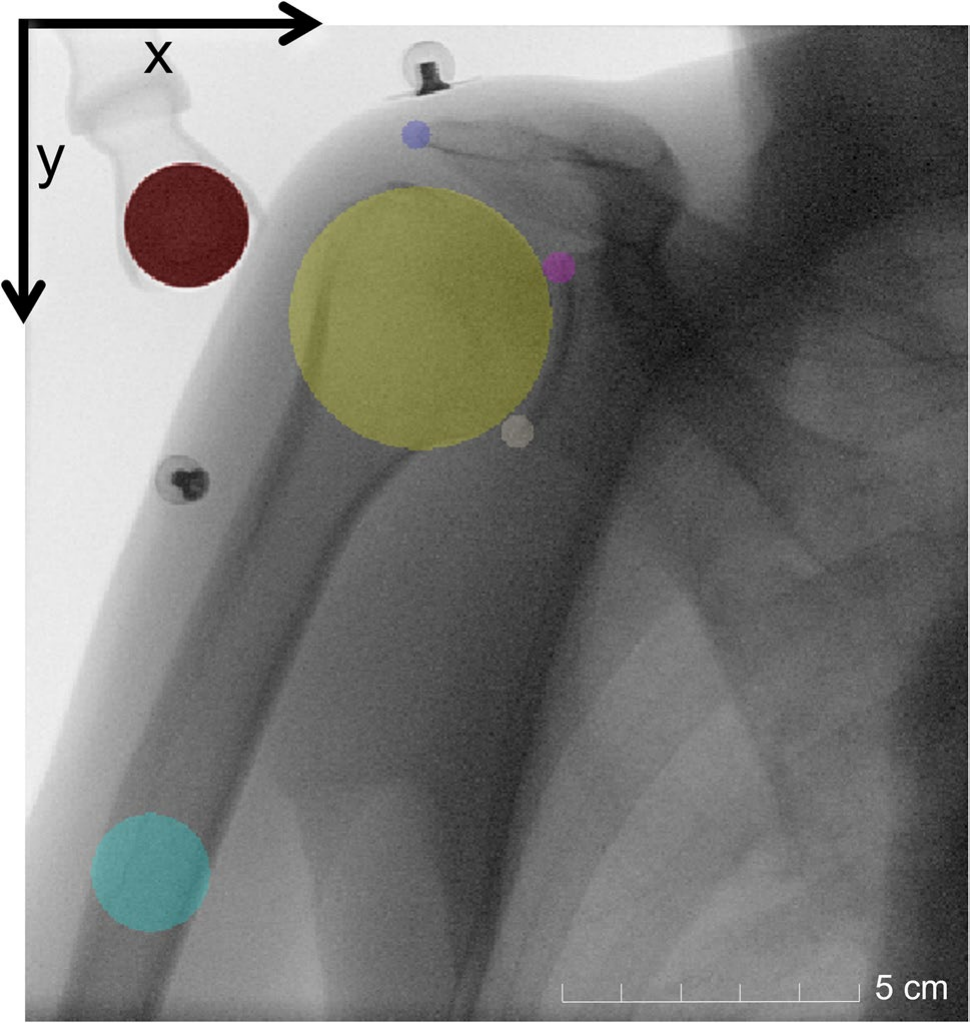
Fluoroscopic image with the mask of the landmarks. Red — reference sphere, yellow — humeral head, blue — lateral point of the acromion, pink — superior edge of the glenoid, white — inferior edge of the glenoid, light blue — humeral shaft

To analyse shoulder kinematics (i.e. scapular rotation, glenohumeral translation or abduction angle) throughout the entire 30° abduction and adduction tasks, five anatomic landmarks must be detected in each fluoroscopic image: the glenohumeral joint centre (defined as the centre of a best-fit circle on the humeral head [1]), the most inferior and superior points on the edge of the glenoid and the most lateral point on the acromion [28] as well as a point on the humeral shaft axis. For calibration purposes, it is also necessary to determine the size of the reference sphere. All landmarks were manually labelled in each image using 3D Slicer [39] (slicer.org) according to the proposed method by Croci et al [28] and were considered ground-truth. The positions of the manually registered landmarks (stored as JSON file) were used to generate a labelled mask with a Python script using the SimpleITK library (simpleitk.org). The mask was created by placing a label disc at the position of each landmark. The discs on the glenoid and acromion had a fixed radius of 8 pixels. The discs of the other landmarks were borrowed by a best fitting circle defined during manual landmark detection [28] (Fig. 2). An nnU-Net [40], a well-established convolutional neural network framework for semantic segmentation of 3D medical images, was trained using fluoroscopic images and disc masks. Pseudo-3D volumes were generated by stacking all fluoroscopic images of an acquisition session together, considering the acquisition timestamp. Shoulders were randomly allocated to training or test sets. The 3D full-resolution network of nnU-Net [40] was trained on 120 fluoroscopic sequences from 40 shoulders (20 subjects) and the corresponding manual landmark segmentations in a fivefold cross-validation process. The data were allocated to these five folds in such a way that all images from the same shoulder were in a single fold. Therefore, the algorithm was never tested on images from shoulders used during training. Each predicted mask was post-processed to remove all but the largest connected component and the landmark coordinates were calculated as the centre of mass for each label in Python and saved as a CSV file. The accuracy of the landmark detection algorithm was tested on 30 unseen fluoroscopic sequences from 10 shoulders (5 subjects) in the test set. If the artificial intelligence network failed to find a label on a fluoroscopic image, it reported a detection failure in that specific fluoroscopic image.

### Data analysis

To evaluate the performance of the semantic segmentation by the trained network, the Dice coefficients between the ground-truth and the predicted masks were reported before deriving the landmark coordinates. The difference between the manually and automatically annotated landmark coordinates was calculated with MATLAB 2021b (The MathWorks). Mean and standard deviation of the distances on the *x*- and *y*-axes of the image coordinate system (i.e. lateral-medial and superior-inferior directions, Fig. 2) and Euclidean distances for each landmark were investigated. Abduction angles were calculated for the manually and automatically annotated images over the entire task, by considering a vertical line and a line passing through the centre of the glenohumeral joint, and the centre of the humeral shaft. The mean and standard deviation of the abduction angle error (absolute difference between manual and automatic sets) were calculated. Moreover, limits of agreement and intraclass correlation coefficients (ICC) were calculated for each landmark coordinate and for the abduction angle over the entire task. In addition, intra-rater and inter-rater analyses of all landmarks were performed on a subset of 20 shoulders (from 10 subjects) from the training set recorded at a sampling rate of 3 Hz.

## Results

Twelve patients with unilateral rotator cuff tear (6 men, 6 women; mean (standard deviation) age: 63.7 ± 9.7 years; height: 174 ± 8 cm; body mass: 78.3 ± 15.6 kg; body mass index (BMI): 26.0 ± 5.7 kg/m^2^) and 13 age-matched control subjects without known shoulder injury (7 men, 6 women; mean (standard deviation) age: 58.2 ± 8.9 years; height: 176 ± 10 cm; body mass: 77.6 ± 13.2 kg; BMI: 25.2 ± 4.7 kg/m^2^) participated in this study. The artificial intelligence network was trained with ground-truth data from 6021 fluoroscopic images of 20 subjects (*n* = 12 with 3Hz and 2118 images, and *n* = 8 with 10 Hz and 3903 images, 40 shoulders, 120 separate sequences) and validated with 1925 fluoroscopic images of 5 subjects (*n* = 1 with 3 Hz and 154 images and *n* = 4 with 10 Hz and 1771 images, 10 shoulders, 30 separate sequences).

**Fig. 3:**
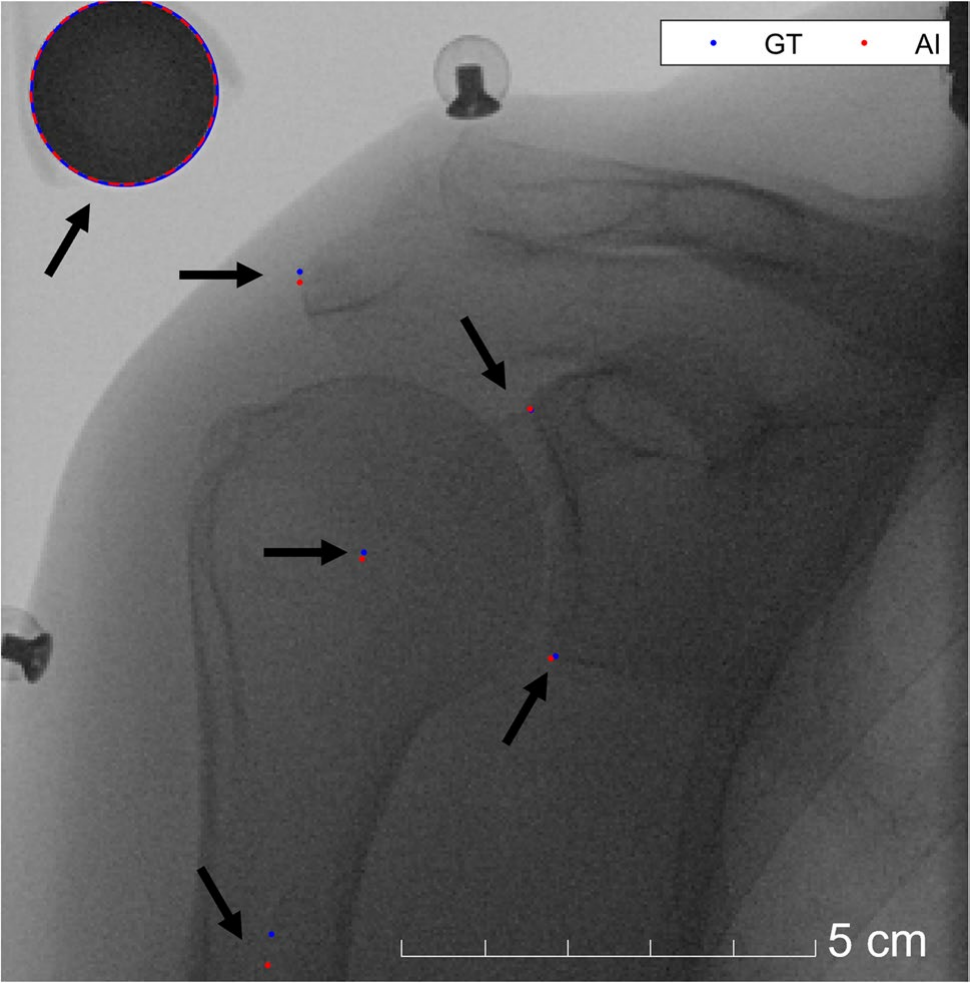
Fluoroscopic image with labelled landmarks. Artificial intelligence (AI) vs. ground-truth (GT). Arrows point the labelled landmarks

Figure 3 shows a fluoroscopic image with the landmarks labelled by artificial intelligence and ground-truth with average accuracy. The nnU-Net framework failed to segment some discs in 264 of 6021 (training) and in 11 of 1925 (test) fluoroscopy images. In the training set, these failed landmark localisations were observed in fluoroscopy images acquired at 3 Hz from eleven shoulders, and at 10 Hz from one shoulder (Table 1). In the latter case, the algorithm completely failed to segment the most lateral point of the acromion. In the test set, the undetected landmarks occurred on eight images acquired at 3 Hz from one shoulder and failed on three images at 10 Hz from two shoulders. The Dice coefficients of the segmented discs used for landmark detection were on average 0.763 ± 0.168 (standard deviation, training) and 0.749 ± 0.182 (test; Table 2).

**Table 1:** Detailed overview of undetected landmarks with artificial intelligence

Landmarks	Number of images with failures of artificial intelligence			
	Training		Test	
	3 Hz (*n* = 2118)	10 Hz (*n* = 3903)	3 Hz (*n* = 154)	10 Hz (*n* = 1771)
Superior edge of glenoid	4	0	0	0
Inferior edge of glenoid	16	0	0	3
Most lateral point of the acromion	21	209	0	0
Glenohumeral joint centre	0	0	0	0
Humeral shaft midpoint	14	0	8	0
Reference sphere	0	0	0	0

**Table 2:** Distances and Dice coefficients of the landmarks between ground-truth and artificial intelligence

Landmarks	Absolute distance on the *x*-axis of the image coordinate system (mm) Mean ± standard deviation		Absolute distance on the *y*-axis of the image coordinate system (mm) Mean ± standard deviation		Euclidean distance (mm) Mean ± standard deviation		Dice coefficients Mean	
	Training	Test	Training	Test	Training	Test	Training	Test
Superior edge of glenoid								
All	0.5 ± 0.5	0.5 ± 0.5	1.0 ± 0.9	1.2 ± 0.7	1.2 ± 1.0	1.4 ± 0.7	0.722	0.678
3 Hz	0.5 ± 0.6	1.1 ± 0.8	1.0 ± 0.9	0.9 ± 0.5	1.3 ± 0.9	1.6 ± 0.7	0.721	0.621
10 Hz	0.5 ± 0.5	0.5 ± 0.4	1.0 ± 0.9	1.2 ± 0.7	1.2 ± 1.0	1.4 ± 0.7	0.722	0.693
Inferior edge of glenoid								
All	0.9 ± 1.0	0.8 ± 0.6	0.8 ± 0.7	0.7 ± 0.6	1.4 ± 1.0	1.2 ± 0.7	0.665	0.714
3 Hz	1.4 ± 1.4	0.6 ± 0.5	1.0 ± 0.9	0.7 ± 0.6	1.8 ± 1.5	1.0 ± 0.7	0.614	0.704
10 Hz	0.7 ± 0.6	0.8 ± 0.6	0.8 ± 0.6	0.7 ± 0.6	1.2 ± 0.7	1.2 ± 0.7	0.728	0.717
Most lateral point of the acromion								
All	0.5 ± 0.5	0.6 ± 0.5	1.2 ± 0.9	1.3 ± 0.9	1.3 ± 1.0	1.5 ± 0.9	0.663	0.648
3 Hz	0.5 ± 0.8	1.2 ± 0.8	1.5 ± 1.2	1.9 ± 0.8	1.6 ± 1.3	2.4 ± 0.9	0.625	0.460
10 Hz	0.4 ± 0.3	0.5 ± 0.5	1.0 ± 0.7	1.3 ± 0.9	1.1 ± 0.7	1.4 ± 0.9	0.709	0.695
Glenohumeral joint centre								
All	0.7 ± 0.5	0.8 ± 0.5	0.7 ± 0.5	0.7 ± 0.4	1.1 ± 0.6	1.1 ± 0.6	0.962	0.954
3 Hz	0.5 ± 0.5	0.9 ± 0.5	0.6 ± 0.5	0.4 ± 0.4	0.9 ± 0.6	1.3 ± 0.5	0.963	0.952
10 Hz	0.8 ± 0.5	0.8 ± 0.5	0.8 ± 0.5	0.7 ± 0.5	1.1 ± 0.6	1.1 ± 0.6	0.960	0.954
Humeral shaft midpoint								
All	2.1 ± 3.1	2.6 ± 2.4	7.1 ± 8.0	8.0 ± 5.4	7.6 ± 8.4	8.6 ± 5.7	0.583	0.520
3 Hz	1.9 ± 2.0	2.0 ± 2.4	6.5 ± 5.3	4.3 ± 3.1	6.9 ± 5.5	4.9 ± 3.7	0.572	0.666
10 Hz	2.2 ± 3.4	2.7 ± 2.4	7.5 ± 9.2	8.3 ± 5.4	8.0 ± 9.7	8.9 ± 5.8	0.602	0.484
Reference sphere								
All	0.1 ± 0.1	0.1 ± 0.0	0.1 ± 0.1	0.1 ± 0.1	0.1 ± 0.1	0.1 ± 0.1	0.983	0.983
3 Hz	0.1 ± 0.1	0.0 ± 0.0	0.1 ± 0.1	0.0 ± 0.0	0.1 ± 0.1	0.0 ± 0.0	0.981	0.983
10 Hz	0.1 ± 0.1	0.1 ± 0.0	0.1 ± 0.0	0.1 ± 0.1	0.1 ± 0.1	0.1 ± 0.1	0.985	0.982

The mean absolute distance on the *x*- and *y*-axes of the image coordinate system and the mean Euclidean distances of the predicted landmark position after extraction from the mask and the manually detected ground-truth landmark position are shown in Table 2. Inaccuracies between artificial intelligence and ground-truth tended to be greater on the *y*-axis and for the images acquired with a pulse rate of 3 Hz. Apart from the humeral shaft midpoint, where the Euclidean distance averaged 9.6 mm in the test set, all other landmarks were located within a distance of 1.5 mm (Table 2). The mean absolute differences in the diameter of the reference sphere were 0.3 ± 0.4 mm (standard deviation) in the training set (3 Hz: 0.3 ± 0.2 mm; 10 Hz: 0.3 ± 0.5 mm), and 0.5 ± 0.1 mm in the test set (3 Hz: 0.4 ± 0.1 mm; 10 Hz: 0.5 mm ± 0.1 mm). The mean absolute differences in the measurements of the abduction angle were 0.8° ± 0.8° in the training set (3 Hz: 1.0° ± 1.0°; 10 Hz: 0.7° ± 0.7°), and 0.9° ± 0.8° in the test set (3 Hz: 0.6° ± 1.3°; 10 Hz: 1.0° ± 0.7°). Overall, the limits of agreement were within 3.1 mm for all landmarks except the humeral shaft midpoint, which had larger values (Table 3 and Table S1 of Supplementary Material). However, the automatic landmarks were consistently placed on the humeral shaft axis, and the limits of agreement were within 2.6° for the abduction angle (Table 4). Excellent reliability was found for all landmarks coordinates (ICC > 0.9), apart from the humeral shaft midpoint along the *y*-axis, which had poor reliability (ICC < 0.2 in the test set, Table 3), but the abduction angle still showed excellent reliability (ICC > 0.9, Table 4).

**Table 3:** Comparison of *x*-axis and *y*-axis landmark coordinates between ground-truth and artificial intelligence of the test set. Shown are mean differences with the limits of agreements, and the intraclass correlation coefficients with 95% confidence interval

Landmarks	Mean difference (mm) [limits of agreement]		Intraclass correlation coefficients [95% confidence interval]	
	*x*-axis	*y*-axis	*x*-axis	*y*-axis
Superior edge of glenoid				
All	0.0 [− 1.4 to 1.4]	− 1.0 [− 2.7 to 0.6]	0.998 [0.998 to 0.998]	0.992 [0.889 to 0.998]
3 Hz	1.1 [0.7 to 2.8]	− 0.8 [− 2.0 to 0.3]	0.994 [0.923 to 0.998]	0.978 [0.578 to 0.994]
10 Hz	− 0.1 [− 1.3 to 1.1]	− 1.1 [− 2.8 to 0.7]	0.997 [0.997 to 0.998]	0.992 [0.882 to 0.997]
Inferior edge of glenoid				
All	0.2 [− 1.7 to 2.1]	0.3 [− 1.5 to 2.1]	0.996 [0.996 to 0.997]	0.995 [0.993 to 0.996]
3 Hz	0.5 [− 0.8 to 1.8]	0.6 [− 0.9 to 2.0]	0.998 [0.992 to 0.999]	0.983 [0.924 to 0.993]
10 Hz	0.2 [− 1.8 to 2.1]	0.3 [− 1.5 to 2.0]	0.994 [0.993 to 0.994]	0.994 [0.993 to 0.996]
Most lateral point of the acromion				
All	0.3 [− 1.1 to 1.7]	1.3 [− 0.5 to 3.1]	0.997 [0.996 to 0.998]	0.993 [0.839 to 0.998]
3 Hz	− 0.5 [− 3.2 to 2.2]	1.8 [0.1 to 3.6]	0.993 [0.989 to 0.996]	0.984 [0.084 to 0.987]
10 Hz	0.3 [− 0.8 to 1.5]	1.2 [− 0.6 to 3.0]	0.997 [0.992 to 0.998]	0.993 [0.859 to 0.998]
Glenohumeral joint centre				
ll	0.0 [− 1.9 to 1.8]	0.1 [− 1.5 to 1.7]	0.996 [0.996 to 0.997]	0.997 [0.997 to 0.998]
3 Hz	− 0.9 [− 2.0 to 0.3]	− 0.7 [− 1.6 to 0.2]	0.997 [0.901 to 0.999]	0.990 [0.674 to 0.997]
10 Hz	0.2 [− 1.8 to 1.8]	0.2 [− 1.4 to 1.7]	0.994 [0.994 to 0.995]	0.997 [0.997 to 0.998]
Humeral shaft midpoint				
All	0.0 [− 7.0 to 7.0]	− 0.4 [− 19.3 to 18.5]	0.970 [0.967 to 0.973]	0.151 [0.107 to 0.194]
3 Hz	1.8 [− 3.3 to 6.9]	4.2 [− 2.2 to 10.5]	0.987 [0.958 to 0.994]	0.775 [0.042 to 0.921]
10 Hz	− 0.1 [− 7.2 to 6.9]	− 0.8 [− 20.2 to 18.6]	0.960 [0.956 to 0.963]	0.092 [0.046 to 0.137]
Reference sphere				
All	0.0 [− 0.1 to 0.1]	0.0 [− 0.2 to 0.2]	1.000 [1.000 to 1.000]	1.000 [1.000 to 1.000]
3 Hz	0.0 [− 0.1 to 0.1]	0.0 [0.0 to 0.1]	1.000 [1.000 to 1.000]	1.000 [1.000 to 1.000]
10 Hz	0.0 [− 0.2 to 0.1]	0.0 [− 0.2 to 0.2]	1.000 [1.000 to 1.000]	1.000 [1.000 to 1.000]

**Table 4:** Mean difference and intraclass correlation coefficients of the abduction angles calculated from ground-truth and artificial intelligence-detected landmarks

Images	Abduction angle			
	Mean difference (°) [limits of agreement]		Intraclass correlation coefficients [95% confidence interval]	
	Training	Test	Training	Test
All	0.0 [− 2.3 to 2.3]	− 0.3 [− 2.6 to 2.1]	0.993 [0.992 to 0.993]	0.991 [0.989 to 0.992]
3 Hz	− 0.3 [− 3.0 to 2.4]	0.0 [− 2.8 to 2.9]	0.991 [0.989 to 0.992]	0.994 [0.991 to 0.996]
10 Hz	0.1 [− 1.8 to 2.1]	0.3 [− 2.8 to 2.9]	0.994 [0.994 to 0.994]	0.990 [0.988 to 0.992]

For the intra- and inter-rater analyses, the subset comprised 1817 fluoroscopic images of the training set (all acquired at 3 Hz). Euclidean distances of the labelled landmarks of this subset are shown in Table 5. The mean absolute differences in the diameter of the reference sphere were 0.2 ± 0.4 mm for intra-rater, 0.3 mm ± 0.2 mm for inter-rater, and 0.3 ± 0.2 mm for artificial intelligence against ground-truth. The mean absolute differences in the measurements of the abduction angle were 0.9° ± 0.7° for intra-rater, 1.3° ± 1.0° for inter-rater, and 0.9° ± 0.7° for artificial intelligence against ground-truth.

**Table 5:** Comparisons of the Euclidean distances for the subset between the different raters

	Euclidean distance for subset (mm) Mean ± standard deviation		
	Intra-rater	Inter-rater	AI vs. GT
Superior edge of glenoid	1.4 ± 0.9	1.5 ± 1.1	1.3 ± 1.0
Inferior edge of glenoid	1.6 ± 1.3	1.8 ± 1.5	1.7 ± 1.5
Most lateral point of the acromion	1.1 ± 0.8	2.0 ± 1.3	1.5 ± 1.3
Glenohumeral joint centre	0.7 ± 0.5	0.8 ± 0.5	0.8 ± 0.6
Humeral shaft midpoint	6.2 ± 5.0	10.2 ± 7.0	7.1 ± 5.6
Reference sphere	0.1 ± 0.1	0.1 ± 0.1	0.1 ± 0.1

AI, artificial intelligence; *GT*, ground-truth

## Discussion

In this study, an artificial intelligence algorithm was developed for the automatic detection of five landmarks (glenohumeral joint centre, a point on the humeral shaft axis, inferior and superior edges of the glenoid and the most lateral point on the acromion) and a calibration sphere on fluoroscopic images acquired with a pulse rate of 3 Hz or 10 Hz. This allows fluoroscopy-based shoulder kinematics to be automatically tracked and analysed in a short time. With this method, the effective radiation dose was at most 0.01 mSv, which is significantly less than that applied for 3D-to-2D model to image registration techniques requiring a CT scan of the shoulder (approximate effective dose 5.8 mSv [41]). This algorithm allows a fast, automatic annotation of fluoroscopic images facilitating computation of the shoulder kinematic parameters such as downward-upward scapular rotation or inferior-superior glenohumeral translation during this 30° arm abduction–adduction task in the scapular plane.

In addition to being faster than manual labelling, this algorithm achieves higher accuracy compared to inter-rater variability and allows for more concise annotation of large datasets. Furthermore, it has the advantage of being deterministic, which means that the same result is always obtained for the same image and in a clinical setting where replicability is critical. This automatic landmark detection algorithm is reproducible for the selected landmarks during an arm abduction and adduction movement. The algorithm detected the landmarks with a low failure rate, failing to find landmarks on only 11 out of 1925 test images (0.57%), and showed greater robustness on fluoroscopic images acquired at 10 Hz than at 3 Hz. This may be because the fluoroscopic images remained consistently sharp during arm movement at faster acquisition rate whereas blurred fluoroscopic images may have occurred in 3-Hz acquisitions. The automatic algorithm annotated all landmarks accurately within a Euclidean distance of 1.5 mm except for the localisation of the humeral shaft midpoint. Because this landmark is solely used for measuring the abduction angle and being defined as a point on the shaft axis, the exact position of this landmark is not well defined. Hence, the manual landmarks are not placed with a consistent distance to the humeral head and therefore impossible to exactly replicate with the automatic algorithm. Despite this inconsistent ground-truth data, the automatic algorithm learned to annotate this landmark consistently on the axis of the humeral shaft leading to an average abduction angle error of 1.0° compared to 1.3° of inter-rater.

The landmark errors of the superior edge of the glenoid and the most lateral point of the acromion were larger along the *y*-axis compared to the *x*-axis. This was also observed with the intra-rater analysis. These landmarks are sometimes more difficult to consistently annotate due to the rounded anatomical edges, and so they are less accurately defined in the inferior-superior direction compared to the mediolateral direction.

Manual image labelling takes about 1 min per image and — depending on the pulse rate at which the images were acquired — accumulating to more than 1 h for labelling an entire sequence acquired at 10 Hz (approximately 80 images per shoulder per abduction–adduction task). In contrast, the automatic landmark detection algorithm can provide sufficiently accurate automatic predictions in less than 1 min for the entire image set. Hence, this automatic landmark detection is potentially suitable for clinically assessing fluoroscopy-based shoulder kinematics. If required, the predicted landmarks may be imported into software for manual correction of the position. Manual correction might be needed in cases where the algorithm fails to find all desired landmarks when the image quality is poor.

In this work, a two-step process for automatic landmark detection is presented by first generating a labelled mask with a 3D deep learning network and then extracting the landmark coordinates from the mask. In a preliminary study, this 3D network outperformed the 2D network (Table S1 of Supplementary Material), even though the third dimension of the stacked fluoroscopy data is in the time domain. Because the fluoroscopy images are acquired periodically, the landmarks always move smoothly from one image to the next. It is believed that, because the 3D kernel also considers the fluoroscopy images before and after the currently processed fluoroscopy image, the 3D network operates in a more robust way in single images of poor quality where certain landmarks would be difficult to find in that particular isolated blurred image. The algorithm presented by Minelli et al [37] outputs the landmark coordinates directly and does not generate an intermediate landmark mask, hence reducing the required computational costs. However, the algorithm is solely applied to single 2D radiographs and interpreting the reliability and accuracy of the landmark position is difficult. The intermediate step of mask generation aids in the interpretation of the landmark localisation process, thereby increasing explainability and robustness [42, 43] and allowing automatic detection of an otherwise failed landmark detection. In the future, an analysis of the variance of the predictions from the nnU-Net subnetworks will be conducted to automatically detect predictions with insufficient accuracy [32].

A limitation of this study is that the algorithm was trained solely with normal shoulders and shoulders with rotator cuff tears, so the performance of the algorithm is likely to decrease in cases where the contours of the glenoid or humerus are not well defined, such as in the presence of severe osteoarthritis. In addition, because the algorithm was trained on single-centre data acquired during a specific movement and always using the same fluoroscopy device, the algorithm presented may require retraining to maintain high performance in landmark detection on data acquired during different movements (e.g. arm abduction of more than 30°) or with different settings, or with other fluoroscopy devices.

In this study, the limited number of participants and the lack of diagnostic images prevented analysis of the data to discriminate between healthy shoulders and shoulders with asymptomatic or symptomatic rotator cuff tears based on kinematics alone. However, the proposed algorithm will facilitate the analysis of kinematic differences analysis in a larger study in the future.

In conclusion, the proposed automatic detection algorithm enables rapid labelling of the fluoroscopic images, especially for images acquired at a sampling rate of at least 10 Hz, allowing tracking and analysis of shoulder motion. Thus, this model facilitates fast measurement of fluoroscopy-based shoulder kinematics during a 30° arm abduction and adduction movement in the scapular plane and thus gaining insight into the kinematics of the shoulder joint in patients with rotator cuff tears.

### Supplementary Information

Below is the link to the electronic supplementary material.

Supplementary file1 (PDF 92 kb)

## Data Availability

The models and algorithms presented in the manuscript, along with the complete results, are available from the corresponding author upon reasonable request.

## Abbreviations

AI: Artificial intelligence
BMI: Body mass index
CT: Computed tomography
GT: Ground-truth
ICC: Intraclass correlation coefficients
MRI: Magnetic resonance imaging

## Declarations

### Guarantor

The scientific guarantor of this publication is Prof. Dr. Annegret Mündermann.

### Statistics and biometry

No complex statistical methods were necessary for this paper.

### Study subjects or cohorts overlap

No study subjects or cohort overlap has been reported.

### Methodology

• prospective

• observational

• performed at one institution
