# Supplementary material for: Fully automatic algorithm for detecting and tracking anatomical shoulder landmarks on fluoroscopy images with artificial intelligence
Source: Eur Radiol. 2023 Aug 11;34(1):270–8. doi: 10.1007/s00330-023-10082-8 (PMC10791975; doi:10.1007/s00330-023-10082-8)
Supplement: Supplementary file 1 — Supplementary file1 (PDF 92 kb) [file 330_2023_10082_MOESM1_ESM.pdf]

Table S1. Comparison of x-and y- landmark coordinates between ground-truth and artificial intelligence of the training set. Shown are mean differences with the limits of agreements, and the intraclass correlation coefficients with 95% confidence interval.

Landmarks		Mean difference (mm)<br>[limits of agreement]		Intraclass correlation coefficients<br>[95% confidence interval]	
		x-axis	y-axis	x-axis	y-axis
Superior edge of glenoid					
	All	0.0<br>[-1.4 – 1.4]	0.1<br>[-2.6 – 2.6]	0.998<br>[0.997 – 0.998]	0.997<br>[0.997 – 0.997]
	3 Hz	-0.1<br>[-1.6 – 1.4]	-0.2<br>[-2.8– 2.4]	0.998<br>[0.997 – 0.998]	0.995<br>[0.995 – 0.996]
	10 Hz	0.1<br>[-1.2 – 1.4]	0.2<br>[-2.5 – 2.9]	0.997<br>[0.997 – 0.997]	0.998<br>[0.997 – 0.998]
Inferior edge of glenoid					
	All	-0.0<br>[-2.7 – 2.6]	-0.1<br>[-2.3 – 2.1]	0.991<br>[0.99 – 0.992]	0.998<br>[0.998 – 0.998]
	3 Hz	-0.3<br>[-4.0 – 3.5]	0.2<br>[-2.4 – 2.7]	0.984<br>[0.982 – 0.986]	0.996<br>[0.995 – 0.996]
	10 Hz	0.0<br>[-1.8 – 1.8]	-0.2<br>[-2.1 – 1.6]	0.995<br>[0.995 – 0.996]	0.999<br>[0.999 – 0.999]
Most lateral point of the acromion					
	All	0.0<br>[-1.4 – 1.3]	0.3<br>[-2.6 – 3.1]	0.998<br>[0.997 – 0.998]	0.997<br>[0.996 – 0.997]
	3 Hz	-0.1<br>[-2.0 – 1.7]	0.6<br>[-4.0 – 2.9]	0.997<br>[0.997 – 0.997]	0.989<br>[0.985 – 0.992]
	10 Hz	0.0<br>[-1.0 – 1.1]	0.8<br>[-1.1 – 2.7]	0.998<br>[0.998 – 0.998]	0.998<br>[0.992 – 0.999]
Glenohumeral joint centre					
	All	-0.1<br>[-1.8 – 1.5]	-0.1<br>[-1.8 – 1.6]	0.996<br>[0.996 – 0.996]	0.999<br>[0.999 – 0.999]
	3 Hz	0.0<br>[-1.5 – 1.4]	0.1<br>[-1.5 – 1.6]	0.998<br>[0.997 – 0.998]	0.998<br>[0.998 – 0.999]
	10 Hz	-0.2<br>[-1.9 – 1.6]	-0.2<br>[-1.9 – 1.5]	0.995<br>[0.9964– 0.995]	0.999<br>[0.999 – 0.999]
Humeral shaft midpoint					
	All	0.6<br>[-6.5 – 7.7]	1.8<br>[-19.0 – 22.5]	0.964<br>[0.961 – 0.967]	0.727<br>[0.707 – 0.745]
	3 Hz	-0.8<br>[-6.1 – 4.5]	3.1<br>[-18.3 – 12.1]	0.970<br>[0.962 – 0.976]	0.675<br>[0.571 – 0.748]
	10 Hz	1.4<br>[-6.1 – 8.9]	4.4<br>[-17.1 – 25.9]	0.959<br>[0.944 – 0.969]	0.729<br>[0.635 – 0.793]
Reference sphere					
	All	0.0<br>[-0.2 – 0.2]	0.0<br>[-0.1 – 0.2]	1.000<br>[1.000 – 1.000]	1.000<br>[1.000 – 1.000]
	3 Hz	0.0<br>[-0.3 – 0.3]	0.0<br>[-0.2 – 0.2]	1.000<br>[1.000 – 1.000]	1.000<br>[1.000 – 1.000]
	10 Hz	0.0<br>[-0.2 – 0.2]	0.0<br>[-0.1 – 0.1]	1.000<br>[1.000 – 1.000]	1.000<br>[1.000 – 1.000]

Table S2. Comparison of 3D vs. 2D networks. Shown are the Dice coefficients for the landmarks of the training set.

Landmarks	Dice coefficients Mean	
	3D network	*2D network*
Reference sphere	0.98	0.96
Glenohumeral joint centre	0.96	0.96
Humeral shaft midpoint	0.58	0.54
Superior edge of glenoid	0.72	0.67
Inferior edge of glenoid	0.67	0.65
Most lateral point of acromion	0.66	0.65
Mean ± standard deviation	0.76 ± 0.17	0.74 ± 0.18
